# A KAP-based evaluation on the role of climate change in shaping dental practices 

**DOI:** 10.6026/973206300211416

**Published:** 2025-06-30

**Authors:** Himanshu Balkumar Gupta, Sibani Sarangi, Rakesh Mutha, Suthar M.B., Akash Ponnukumar, Vikram Karande, Ritik Kashwani

**Affiliations:** 1 CSMSS Dental College and Hospital, Director, Mahaveer Multi-speciality Hospital, Chhatrapati Sambhajinagar, Maharashtra, India; 2 Department of Periodontology, Hitech Dental College and Hospital, Bhubaneswar, India; 3 Department of Periodontology, DY Patil Dental School, Pune, Maharashtra, India; 4 Department of K. K. Shah Jarodwala Maninagar Science College, Ahmedabad, Gujarat, India; 5 Department of Orthodontics, Indira Gandhi Institute of Dental Sciences, Sri Balaji Vidyapeeth (deemed to be) University, Puducherry, India; 6 Department of Oral and Maxillofacial Surgery, D.Y. Patil Dental School, Lohegaon, Pune, Maharashtra, India; 7 Department of Oral Medicine and Radiology, School of Dental Sciences, Sharda University, Greater Noida, Uttar Pradesh, India

**Keywords:** Climate change, dentistry, knowledge-attitude-practice (KAP), oral health, sustainability

## Abstract

Climate change poses a significant threat to oral health and the sustainability of dental care systems. Therefore, it is of interest to assess the dental students' knowledge, attitudes and practices (KAP) on climate change and sustainability. While most understood the environmental impact and felt responsible, fewer individuals adopted sustainable practices, such as maintaining digital records and segregating waste. Barriers included a lack of training and institutional support. The findings underscore the need for curriculum reforms and policy adjustments to foster sustainability in dentistry.

## Background:

Climate change is recognised as one of the primary global health issues of the 21st century. Its impact extends far beyond environmental degradation, affecting public health, infrastructure, disease epidemiology and access to healthcare services in profound ways [1]. Compared to numerous healthcare branches, dentistry has received relatively little attention regarding climate change, despite its high environmental impact and exposure to climate-related disruptions [2, 3]. An increase in temperature, water shortages, air pollution and a rise in the frequency of extreme weather events all contribute, directly or indirectly, to the increase in oral health risks. Dehydration, changes in diets resulting from food insecurity, shifts in disease patterns and a rise in stress levels are all associated with adverse oral health outcomes [4, 5]. At the same time, environmental changes can cause disruptions in dental care delivery through damage to infrastructure, disruption of the supply chain and a lack of utilities [6]. Furthermore, climate-sensitive diseases, such as vector-borne infections, may exhibit oral manifestations, underscoring the need to implement integrated dental care in climate-prone areas [7]. At the same time, environmental deterioration is also a contributor to dental health issues. Dental practices have a high energy demand, use a significant amount of water and generate substantial amounts of single-use plastics, as well as biomedical and chemical waste. The enhanced use of infection control protocols, especially after the COVID-19 pandemic, has heightened consumption of disposable materials, worsening environmental concerns [8]. Despite these ties, climate change remains a fringe subject in dental education and debate. The incorporation of sustainability into the dental curriculum is still in its development and only a few healthcare professionals get training in eco-conscious dental practices [9]. It is important to know how the future dental professionals see the question; and whether they are ready to act sustainably in order to close this gap. Therefore, it is of interest to assess knowledge, attitudes and practices (KAP) among dental students and interns, with regard to climate change and dentistry.

## Methodology:

## Study design and setting:

This study collected information from dental students and interns to determine their perceptions and understanding of the impact of climate change on dentistry. In April 2025, the study was carried out in India.

## Study population and sample size:

The research covered BDS degree students, from the second year ahead, as well as students in their internship. The study included 123 people who expressed interest and were available at the time. The number of cases analyzed was deemed sufficient to describe the trends and proportions of the given population.

## Inclusion and exclusion criteria:

## Inclusion criteria:

[1] In the second, third and final years, BDS students are included.

[2] Interns undergoing clinical training.

[3] Participants who provided informed consent.

## Exclusion criteria:

[1] Students absent during data collection.

[2] Incomplete or invalid responses.

## Data collection tool:

The process of collecting data depended on a planned and tested questionnaire. Experts and a significant amount of literature were used to design the questionnaire. It comprised four sections: Information relating to students' age, gender and year in school. The Knowledge Section consists of 6 multiple-choice questions that focus on the effect of climate change on oral health and dentistry. Attitude Section - 5 questions are given using a 5-point Likert scale (strongly disagree to strongly agree) that ask about beliefs and sense of responsibility. Practice Section - There are five questions looking at whether sustainable practices are used in clinical or academic settings (yes/no/not sure). A team of three experts in education reviewed the questionnaire and it was tested on a group of 10 students (not taken into account in the final study) to guarantee that language was clear and reliable.

## Data collection procedure:

One week was spent in collecting the data from both class and clinical scenarios. Each questionnaire was given out as a hard copy and completed while being supervised, preventing others from influencing the answers. All components were checked for lacking immediately after arrival.

## Data analysis:

Entered the data into SPSS version 26.0 (IBM Corp., Armonk, NY, USA) and did the analysis. Participant information and knowledge, attitudes and practices were summed up using descriptive statistics like frequencies, percentages, means and standard deviations. To determine the connection between KAP scores and demographic factors, the Chi-square test and t-tests were used, where the significance level was set to p < 0.05.

## Ethical considerations:

The Institutional Ethics Committee of the dental college approved this study. Taking part was not required and all respondents gave their written agreement. Participants were made sure their privacy was always preserved during the study.

## Results:

A total of 123 participants completed the questionnaire. Of these, 72 (58.5%) were male and 51 (41.5%) were female. A total of 123 participants completed the questionnaire, with 72 (58.5%) males and 51 (41.5%) females. Regarding knowledge, 98 (79.7%) were aware that climate change can lead to oral health problems such as dehydration and infections, while 25 (20.3%) were unaware. A majority of 84 participants (68.3%) recognized that dental practices contribute to greenhouse gas emissions and pollution, whereas 39 (31.7%) were unsure or unaware. Most respondents, 110 (89.4%), agreed on the importance of proper dental waste management for environmental protection, with only 13 (10.6%) disagreeing. Over three-quarters understood that climate-related water shortages affect dental hygiene practices. Additionally, around 60% acknowledged that rising temperatures can increase dental emergencies or oral stress disorders, though nearly 40% did not. Lastly, 87 participants (70.7%) knew that environmentally sustainable dental methods help reduce environmental impact, while 36 (29.3%) lacked this awareness (Table 1 - see PDf). People were not all in agreement about how dangerous climate change is to our health. A very small number of participants (3.3%) strongly believed that the statement was false and 5.7% disagreed, suggesting a reluctance to accept the health risks of climate change. Nearly 14% of those surveyed stayed neutral and did not give either a yes or no answer. However, most individuals agreed with this statement; 49.6% of them felt it represented a serious threat and 27.6% were very convinced about it. Users were asked if they thought dentists ought to play a part in sustainability. A little over 2% strongly disagreed and close to 8% disagreed, reflecting that not many think that dentistry includes caring for environmental issues. Almost 15% of the group answered neutrally, which could be due to being unsure of their duties regarding this subject. On the positive side, 48% agreed and 26.8% strongly supported the idea of dentists promoting ways to be eco-friendly.

When asked about making dental clinics eco-friendly, most participants agreed, as 0.8% strongly disagreed and 4.9% disagreed, with very few participants being against the idea or not sure about it. It seemed that over 18% of the people surveyed were neutral, indicating that they were not aware of how eco-friendly setups work. Furthermore, 47.2% of them believed that green practices should be used in dentist offices, with an additional 28.5% strongly agreeing, which means that the majority supported green actions in clinics. In terms of whether teaching environmentally friendly oral habits to patients is necessary, 53.7% disagree slightly or strongly, suggesting dentists are disputing their role as educators on this matter. More than half (59.9%) of the respondents agreed and 39.4% strongly agreed that teaching patients about sustainable practices is an important duty for healthcare workers, so most of the group supported this idea. People had more differing views about whether adopting sustainable dental practiced impact the quality of care. Of the participants, 9.8% strongly disagreed and 17.1% disagreed, meaning that these people thought safe practices would not improve the quality of the treatment. In addition, 29.3% chose the middle answer, suggesting they did not know much about sustainable technologies. Another group of dentists (31.7% of them) agreed, while 12.2% adopted a stronger stance, showing that they have doubts about how sustainability could influence standard or efficiency in dental care (Table 2 - see PDF). Of the 123 respondents, 54 (43.9%) reported using digital records, indicating a shift towards paperless documentation, while 58 (47.2%) still relied on paper charts. Eleven participants (8.9%) were unsure about their use of digital or paper records. Only 32 participants (26.0%) stated they had received formal training on sustainability in dentistry, whereas 79 (64.2%) had not and 12 (9.8%) were unsure if such training was provided. Regarding dental waste segregation, 65 respondents (52.8%) followed proper waste management practices, 44 (35.8%) did not and 14 (11.4%) were uncertain. For energy-efficient or water-saving dental equipment, 45 participants (36.6%) used such technologies, 62 (50.4%) did not and 16 (13.0%) were unsure. When asked about encouraging patients to adopt eco-friendly oral habits, 50 (40.7%) answered "Yes," 58 (47.2%) said "No," and 15 (12.2%) were uncertain. These figures reflect partial adoption of sustainable practices alongside significant gaps in knowledge, resources and training (Table 3 - see PDF).

## Discussion:

The findings from the KAP questionnaire regarding the impact of climate change on dentistry are supported by research from international sources on the subject. Similar to Hackley *et al.* [10] and Bhadauria *et al.* [4] 79.7% of participants in this research realized that climate change can affect oral health. According to surveys, more than 70% of dental professionals believe it is important for dental healthcare providers to consider sustainability. Still, researchers noticed that only a portion of individuals employed digital records, energy-efficient equipment, or took part in planned training for sustainability, which suggests that some individuals are not acting on their awareness. Likewise, Nassar *et al.* [11] noticed while people understood and were positive about the topic, the lack of support at work and school stopped them from making continued changes. Additionally, a majority of the dentists here reported receiving little to no formal education on eco-friendly dentistry, which confirms earlier studies by Kalra *et al.* [9] and Duane *et al.* [12]. Furthermore, 52.8% of the respondents disposed of their waste correctly, which suggests that while the adoption of environmentally conscious methods is commendable, it is still inadequate compared to results worldwide, according to Sanguida *et al.* [13] and Tawade *et al.* [14]. The global climate crisis is reshaping all sectors, including healthcare and dentistry. As weather patterns become more volatile and environmental health determinants grow, the principles of Environmental, Social, and Governance (ESG) are now essential for healthcare organizations. Dental practices must assess their ecological footprint, social responsibility, and governance for sustainable operations. Climate change is already impacting patient demographics, disease patterns, material sourcing, and operational resilience in dentistry, urging innovation and adaptation for a sustainable future in oral health [15].

Research highlights the importance of environmental care in dentistry. One document on the subject studied commuting emissions in a dental school and recommended implementing green transport policies and electronic alternatives. The disposal of waste material from gypsum items is another issue in prosthodontics. Methods of recycling waste reduce landfill waste and help save on costs. Still, being more eco-friendly is not widely practiced by dentists, as there can be obstacles in terms of finances, education and access to proper equipment. It isn't easy to include sustainability in dental education because educators may not be sufficiently qualified and there is not enough time in the curriculum. To progress in sustainability, dentists, teachers and members of every dental institution should join efforts. Although the participants were concerned about sustainability and prepared to support it, the dental practice activities remained limited. As a result, updated curricula, proper training and support from the school are needed to help close this information gap.

## Conclusion:

Dental students and interns have strong knowledge and a supportive attitude toward sustainable oral health care. Still, they do not consistently demonstrate the actions that reflect these beliefs. Communities of dentists around the world can tackle climate change and offer top-quality patient care by raising awareness on environmental matters and giving future dental workers the tools they need. Implementing institutional support and effective policies is crucial for green dentistry to continue and thrive in all areas.
